# Comparing aerobic and resistance exercise emphasis during androgen deprivation and radiation therapy for prostate cancer: A randomised feasibility trial

**DOI:** 10.1007/s00520-025-09650-z

**Published:** 2025-06-20

**Authors:** Kira Murphy, Bróna Kehoe, Suzanne Denieffe, Dayle Hacking, Ciaran M. Fairman, Michael Harrison

**Affiliations:** 1 Department of Sport and Exercise Science, South East Technological University, Cork Rd, Waterford, Ireland; 2 UPMC Hillman Cancer Center, Whitfield Hospital, Waterford, Ireland; 3 School of Humanities, South East Technological University, Waterford, Ireland; 4 Department of Exercise Science, Arnold School of Public Health, University of South Carolina, Columbia, SC USA

**Keywords:** Prostate cancer, Androgen deprivation therapy, Radiation therapy, Quality of life, Exercise oncology

## Abstract

**Purpose:**

Most exercise interventions for men with prostate cancer utilise resistance and aerobic exercise, though the optimal combination of each for cardiometabolic health and quality of life outcomes is unclear. This study aimed to determine the feasibility of an aerobic-emphasised (AE) versus a resistance-emphasised (RE) exercise intervention in men with prostate cancer undergoing androgen deprivation therapy (ADT) and radiation therapy (RT).

**Methods:**

A 6-month two-armed randomised feasibility study was undertaken. Prostate cancer patients (*n* = 24) undergoing ADT and RT were randomised to either an AE (*n* = 12) or RE (*n* = 12) supervised programme. The primary outcome was feasibility, assessed via recruitment, retention, adherence and safety.

**Results:**

Twenty-four men were randomised, the recruitment rate was 19%. For AE and RE respectively, retention was 75% and 83%, adherence to the exercise prescription was 80% and 76%, attendance was 91% and 92%, with attendance during RT at 96% and 95%. No serious adverse events were recorded. Preliminary evidence favoured the AE intervention (*p* < 0.05) for certain quality of life domains and haematology markers and the RE intervention (*p* = 0.05) for BESS balance scores. Pre- to post-intervention improvements (*p* < 0.05) were observed in multiple functional fitness outcomes.

**Conclusion:**

An exercise trial that carefully varies both resistance and aerobic elements is feasible for men with prostate cancer undergoing active treatment. Strategies would have to be implemented to increase recruitment for a larger trial.

**Trial registration:**

The trial has been registered on ClinicalTrials.gov as of the 14^th^ of December 2021 (NCT05156424).

## Introduction

Androgen deprivation therapy (ADT) in conjunction with radiation therapy (RT) is a common treatment for intermediate and high-risk prostate cancer. This therapeutic approach is favoured among older men, men with poorer performance status and unfavourable disease characteristics over surgical intervention [1], as both have comparable results with regards to prostate-cancer-specific mortality and disease progression [2]. However, the adverse side effects of ADT and RT are extensive and include alterations in body composition, diminishing physical function, rectal bleeding, urinary incontinence, erectile dysfunction, heightened psychological distress and increased fatigue [3–5]. One of the most concerning effects of ADT is its potential effects on cardiovascular disease risk, including an increased risk of coronary heart disease, myocardial infarction and sudden cardiac death [6]. Additionally, studies have indicated a reduction in insulin sensitivity and elevated plasma insulin levels during ADT treatment [6, 7], contributing to a rise in metabolic disorders among those treated [8]. With a 10-year relative survival rate of 98% [9], there is a growing population of men enduring treatment-related side effects, concurrent with other comorbidities on a long-term basis, leading to a deterioration in their overall quality of life. There is an increasing demand for supplementary therapies aimed at alleviating the adverse effects experienced by men treated for prostate cancer.

Exercise has shown promise with regards combating the side effects of prostate cancer and its treatment. Existing literature demonstrates safety and reports multiple benefits from exercise for participants [10–15]. Most studies to date have utilised a combined aerobic and resistance exercise intervention [16–20] or a resistance alone intervention [21–24] with comparison to a control group. Reported benefits include enhanced quality of life, fatigue, cardiovascular fitness, functional fitness and lean mass, with mixed outcomes with regards fat mass [25]. Currently, only a limited number of studies have directly compared resistance versus aerobic exercise modalities, reporting similar outcomes in terms of quality of life and fatigue [26–29]. In a real-world setting, exercise programmes are unlikely to be exclusively aerobic or resistance alone, and various combinations of both modalities may offer the most suitable prescription. Given the diverse side effects of ADT and RT, arguments could be made for aerobic-focused programmes to address fatigue, stress, fat reduction, and cardiovascular risk [30, 31], while resistance-focused programmes could be argued for bone density, strength and muscle loss [21, 22, 24, 32, 33]. Both resistance and aerobic exercises may be beneficial with regards insulin sensitivity, blood glucose control and quality of life improvements [34, 35]. As a further complication, Newton and colleagues [33, 36] raise the possibility of an interference effect from aerobic exercise, compromising hypertrophy gains from resistance exercise. They suggest that aerobic exercise may need to be limited or avoided in the presence of ADT [36] but also a need for further comparison studies to elucidate these complexities and the optimal exercise prescription [33].

A randomised controlled trial is warranted to compare the efficacy of a resistance-emphasised (major component resistance, minor component aerobic) versus aerobic-emphasised (major component aerobic, minor component resistance) intervention in mitigating the side effects of ADT and RT. Such trials need to provide appropriate exercise stimuli with progressive overload of both the aerobic and resistance element in order to optimise potential gains. Designing such a multicomponent trial poses inherent complexities, with a need to mix high and moderate intensities of resistance and aerobic exercise in each trial arm and progress each over time. Complexities are increased in the case of participants who are commencing ADT treatment with a block of RT scheduled during the exercise intervention. The literature contains multiple trials of exercise during ADT treatment [16–24, 29, 31, 32] but few [26, 27, 37, 38] where there is clear reporting of RT for the majority of participants during the intervention period. In line with the MRC Framework and Consort 2010 Statement [39, 40], a feasibility study is justified to identify practical challenges and consider the ability of participants to comply with the challenging programme design during the treatment period.

The aim of this study was to therefore to determine the feasibility of a trial to compare an aerobic-emphasised (AE) with a resistance-emphasised (RE) exercise intervention in men with prostate cancer undergoing ADT and RT. Preliminary evidence of difference between trial arms was investigated by assessing cardiometabolic health, fitness and quality of life outcomes.

## Methods

### Study design

This two-armed randomised feasibility trial compared an aerobic-emphasised (AE) to a resistance-emphasised (RE) exercise intervention aimed at improving cardiometabolic health and quality of life parameters in men with prostate cancer undergoing ADT and RT. Ethical approval was obtained from the HSE South-East Area Research Ethics Committee and Waterford Institute of Technology (WIT) Research Ethics Committee. This trial was registered on ClinicalTrials.gov (NCT05156424). Participants were recruited through a single hospital site in the South East of Ireland. Eligible participants were identified by a radiation oncologist at a consultation visit, the majority (70%) at the first consultation post-diagnosis prior to commencement of treatment, the remainder at the post-RT consultation. Subsequently, interested participants received a study information leaflet to review with follow up from the trial coordinator 48 h later. Reasons for declining to participate were recorded at this point. All participants provided informed consent before data collection. A detailed trial protocol has been published [41] and is summarised below.

### Participants

Men diagnosed with intermediate or high-risk prostate cancer, who were undergoing ADT with curative intent were invited to take part in the study. Intermediate risk is defined as a tumour confined to the prostate, prostate specific antigen (PSA) 10–20, or Gleason score of 7. High risk is defined as a tumour extending outside of the prostate, PSA > 20 or Gleason score ≥ 8. Participants had to be at least 18 yrs old with a histologically diagnosed prostate cancer, prescribed ADT, self-reporting not to be taking part in regular structured exercise and medically cleared to exercise by their oncologist. Exclusion criteria included prior exposure to ADT > 12 months, prior hypogonadism, established metastatic bone disease or established osteoporosis and any medical condition that could put them at risk from exercise as judged by their attending oncologist. Of the 24 recruited into the study, 19 entered at the start of ADT and subsequently were also treated with RT, the remaining 5 were recruited after RT.

### Randomisation

Following completion of baseline testing, participants were randomly assigned to the AE or the RE group. Randomisation occurred in a ratio of 1:1, with stratification by intermediate vs high risk prostate cancer. A research statistician, independent to the study and blinded to identifying information and assessment results generated the random computerised number sequence using block sizes of four.

### Exercise intervention

Both groups attended twice weekly supervised sessions for 24 weeks. Participants were offered 1-to-1 or small group sessions at a time and on a day that was convenient for them, with group size limited to 5. The initial phase of the exercise intervention focused on safe exercise practices and determined appropriate individual training loads. Subsequent phases emphasised progressive and autoregulated exercise blocks. All sessions were approximately 60 min in duration. Both groups performed the same warm-up and cool down, with the remaining time (40 min) divided between the primary emphasised exercise mode (30 min) and the secondary exercise mode (10 min), hence a 3:1 ratio based on time. Session 1 of the week focused on lower intensity/higher volume exercises for both the aerobic and resistance components while session 2 focused on higher intensity/lower volume exercises.

The aerobic element of the intervention consisted of both moderate intensity continuous training (MICT) and interval (high intensity) training. Exercise intensity was adjusted using the modified Borg Rate of Perceived Exertion (RPE) scale. The resistance element utilised a daily undulated periodisation approach, focusing on hypertrophy work and was progressed using the “2 for 2” rule [42]. In the case of pin-loaded machines, the pin was moved to the next available weight.

A home-based component was introduced in week 19 to supplement the supervised sessions, increasing exercise volume without adding travel or resource burden on participants. The home element, consisting of one additional 30-min session weekly aimed to mimic the supervised sessions. Participants received specific aerobic or resistance workouts based on their assigned groups with body weight movements, resistance bands and household items used for resistance activity. Completion of these home-based sessions was recorded at the subsequent supervised session and confirmed at the end of the trial based on self-report. Full details of the exercise intervention have been described previously [41].

### Outcome measures

#### Feasibility outcomes

Feasibility was determined via assessment of recruitment, retention, adherence, safety, suitability of the testing protocols and acceptability. The comparison of the AE and RE group changes, albeit with small sample sizes, may be considered a further feasibility domain [43]. Recruitment was assessed by calculating recruitment rate (% of eligible patients that were randomised to the study) and reporting the reasons why eligible patients declined to participate. Retention was reported as the percentage of randomised participants to complete the post intervention assessments. The number of dropouts and the reasons for same were reported for each group. Adherence was assessed through several conventional and exploratory methods as recommended for clinical exercise trials [44]. This included attendance rate (% of attended supervised sessions compared to scheduled sessions), exercise interruption frequency (missing at least three consecutive supervised sessions), programme paused frequency (programme electively paused but missed sessions added to end of the programme), early session termination frequency and adherence to the exercise prescription (% of sessions where no element of a session required negative dose modification). Adherence to the resistance exercise prescription is also expressed in terms of exercise relative dose intensity [45]. Safety was assessed via the number and severity of any adverse event concerning the exercise programme or testing sessions and was reported using the common terminology criteria for adverse events (CTCAE) v5.0 grading system [46]. Where it was not possible for a participant to undertake one of the outcome measure testing protocols, the reason was noted. Acceptability was assessed through qualitative interviews at the end of the intervention, the results of which have been published previously [47].

#### Fitness, cardiometabolic health and quality of life outcomes

A range of fitness, cardiometabolic health and quality of life outcomes were assessed at the pre- and post-intervention timepoints. A limited interim assessment was also completed at week 7 to gauge the initial response to the exercise intervention prior to the commencement of RT. Participants performed two familiarisation sessions prior to performing any fitness assessments at baseline. Quality of life was assessed through the Medical Outcomes Short Form 36 (SF-36) [48], European Organisation for the Research and Treatment of Cancer Quality of Life Questionnaire (EORTC QLQ) C-30 [49] and EORTC QLQ-PR25 [50] for general health-related QOL, cancer specific QOL and prostate cancer specific QOL respectively. Fatigue was assessed through the Functional Assessment of Cancer Therapy-Fatigue (FACT-F) [51]. Vascular health was assessed via systolic and diastolic blood pressure (Omron, Omron Healthcare, UK) and pulse wave velocity (Complior Analyse, Alam Medical, France). Anthropometrics and body composition were assessed via whole-body and regional measurements for lean soft tissue and fat mass using dual energy x-ray absorptiometry (DEXA) (Horizon W, Hologic Inc., Waltham, MA), as well as Body Mass Index (BMI) calculations. Aerobic fitness was assessed through a number of validated measures including a submaximal incremental treadmill test employing a modified Bruce protocol [52] and six-minute walk test [53]. Strength assessments involved a one-repetition maximum evaluation for both leg press and chest press, conducted using a Precor seated leg press machine and seated chest press machine, respectively (Precor Incorporated, Woodinville, Washington, USA). Knee extension and flexion peak torque were also assessed using isokinetic dynamometry (Biodex System 4 Model 835–210, Shirley, New York, USA), following a protocol used previously [54]. Additionally, a grip test was performed using a dynamometer as an assessment of upper body strength [55]. Leg muscle endurance was evaluated using the 30-s sit-to-stand test (STS) [56]. Mobility, balance and flexibility were assessed using the timed up and go test (TUG) [57], Balance Error Scoring System (BESS) [58] and a sit and reach test [59] respectively. The Godin Leisure Time Exercise questionnaire was used to evaluate physical activity levels [60]. Metabolic health markers and haematological markers were determined in the fasted state. These included circulating lipids, glucose, insulin, C-reactive protein and full blood count parameters. Full details on all measures have been described previously [41].

### Statistical Analysis

Pre-intervention AE and RE group values were compared using independent t-tests or Chi-Square as appropriate. To assess between group intervention differences, the pre- to post-intervention change scores in the AE and RE groups were compared using independent t-tests. Changes in the combined AE and RE groups (*n* = 19) over time, potentially reflecting a generalised effect of exercise or an effect of treatment, were analysed using paired t-tests or a one-way repeated measures Analysis of Variance (for outcomes with interim assessments). The effect size for these pre- to post-intervention differences is expressed as Cohen’s d [61]. Significance was set at *p* < 0.05. Values are presented as mean ± standard deviation (SD).

To assist with sample size calculations for future larger trials, test- retest Pearson correlation coefficients were calculated using the pre- and post-intervention values of the combined group (*n* = 19). Sample size estimates for future trials were based on an Analysis of Covariance (ANCOVA) approach to analysis with adjustment for the test–retest correlation coefficient) [62].

## Results

### Recruitment and retention

**Fig. 1:**
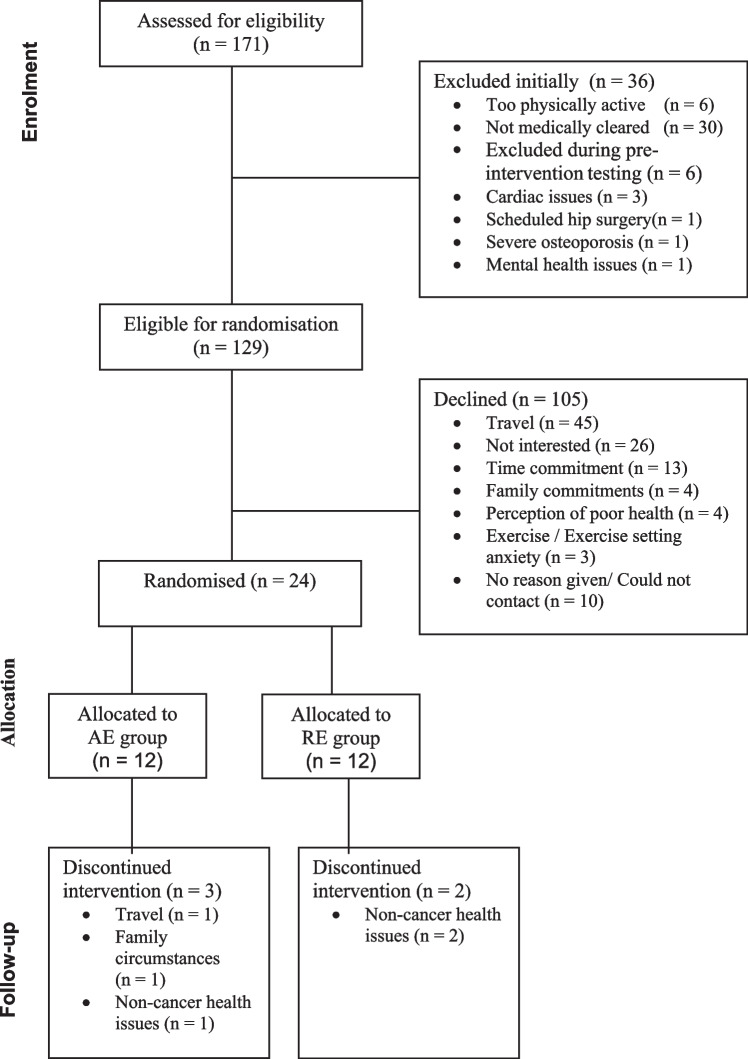
Consort diagram

Recruitment took place between August 2021 and October 2022. Out of a total of 171 intermediate to high-risk patients who were referred for RT and ADT, 135 met eligibility criteria and were medically cleared to take part in the programme, though 6 were subsequently excluded during testing so that 129 (75%) were eligible for randomisation. Twenty-four patients agreed to participate and were randomised to either the AE or RE groups, giving a recruitment rate of 19%. Of these, 19 were commencing treatment and 5 (AE *n* = 2, RE *n* = 3) had just completed RT. The main reason given for declining to participate was travel burden to the exercise centre (43%), followed by a lack of interest (25%). Retention rate in the programme was 75% and 83% for AE and RE respectively (79% for the combined group). Five participants withdrew from the programme, three due to health issues unrelated to the exercise intervention or their prostate cancer, one due to the travel commitment and one due to a change in family circumstance. More details on participant flow can be found in the Consort Diagram (Fig. 1).

### Participant characteristics

The average age of those randomised was 71.2 ± 6.8 years (range 59–82 years). Of the 24 randomised, 67% had hypertension, 25% had ischemic heart disease and/or atrial fibrillation and 33% had diagnosed arthritis. The pre-intervention characteristics of the AE and RE groups are shown in Table 1. There were no sociodemographic or treatment differences between the groups at baseline (Table 1). There were no physical function, body composition, vascular, haematological or metabolic differences between the groups at baseline (Table 3). Some differences were observed at baseline with respect to FACT-F and selected quality of life domains in both the EORTC-QLQ-30 and SF-36 instruments (Table 3).

**Table 1:** Pre-intervention characteristics of all participants who were randomised (*n* = 24) and those who completed the aerobic-emphasised (*n* = 9) and resistance-emphasised (*n* = 10) arms

Pre-intervention variables	Randomised (*n* = 24)	Aerobic- Emphasised (*n* = 9)	Resistance- Emphasised (*n* = 10)	*P* value
Age (years)	71.2 (6.8)	70.2 (4.9)	70.1 (8.3)	0.97
Weight (kg)	86.1 (16.5)	82.4 (16.0)	87.1 (12.1)	0.48
Height (cm)	171.8 (6.8)	172.8 (6.3)	169.9 (6.1)	0.31
Body Mass Index (kg/m^2^)	29.1 (4.3)	27.5 (4.5)	30.2 (3.5)	0.16
Education, *n* (%)				0.80
* Primary*	11 (46%)	5 (56%)	4 (40%)	
* Secondary*	6 (25%)	2 (22%)	3 (30%)	
* Tertiary*	7 (29%)	2 (22%)	3 (30%)	
Employment, *n* (%)				0.96
* Retired*	13 (54%)	5 (56%)	5 (50%)	
* Employed (part-time)*	9 (38%)	3 (33%)	4 (40%)	
* Employed (full-time)*	2 (8%)	1 (11%)	1 (10%)	
Marital status, *n* (%)				0.39
* Married*	16 (67%)	6 (67%)	7 (70%)	
* Divorced*	5 (21%)	3 (33%)	1 (10%)	
* Widowed*	2 (8%)	0 (0%)	1 (10%)	
* Single*	1 (4%)	0 (0%)	1 (10%)	
Health Behaviours				
Current smoker, *n* (%)	3 (13%)	3 (33%)	0 (0)	0.09
Alcohol consumer, *n* (%)	22 (92%)	9 (100%)	8 (80%)	0.47
Godin Leisure-Time Exercise score	7.1 (7.6)	7.5 (8.1)	6.8 (7.6)	0.85
Private health insurance, *n* (%)	8 (33%)	4 (44%)	4 (40%)	1.0
Co-morbidities				
Number of medications	2.9 (2.3)	2.6 (1.8)	2.6 (2.5)	0.97
Number of comorbidities	1.6 (1.2)	1.6(1.1)	1.1 (1.1)	0.39
Chronic obstructive pulmonary disease, *n* (%)	4 (17%)	2 (22%)	1 (10%)	0.58
Diabetes, *n* (%)	0 (0%)	0 (0%)	0 (0%)	
Cardiovascular disease (ischemic heart disease & atrial fibrillation), *n* (%)	6 (25%)	1 (11%)	2 (20%)	1.0
Hypertension, *n* (%)	16 (67%)	6 (67%)	5 (50%)	0.65
Dyslipideamia, *n* (%)	12 (50%)	5 (56%)	3 (30%)	0.37
Arthritis, *n* (%)	8 (33%)	2 (22%)	4 (40%)	0.63
Prostate cancer risk				
Gleason score	7.5 (0.7)	7.6 (0.5)	7.6 (0.8)	0.89
Prostate cancer risk, *n* (%)				0.65
* Intermediate*	9 (38%)	4 (44%)	3 (30%)	
* High*	15 (62%)	5 (56%)	7 (70%)	
Radiation therapy				
Radiation therapy during exercise intervention, *n* (%)	19 (79%)	7 (78%)	9 (90%)	0.58
Number of radiation therapy treatments, mean	26.1 (7.5)	24.3 (6.0)	28.8 (8.1)	0.20
Radiation therapy dose (Gy)	65.6 (8.0)	65.5 (6.1)	66.8 (9.9)	0.75
Androgen deprivation therapy type				
* Anti-androgen (Bicalatamide), n (%)*	24 (100%)	9 (100%)	10 (100%)	
* Luteinising hormone-releasing hormone agonist (Triptorelin) n (%)*	24 (100%)	9 (100%)	10 (100%)	
ADT duration prior to study entry, *n* (%)				0.22
< *2 months*	18 (75%)	6 (67%)	9 (90%)	
> *2 months*	6 (25%)	3 (33%)	1 (10%)	

Values are mean (SD) or *n* (%). *P* value for the pre-intervention comparison of the aerobic-emphasised and resistance-emphasised groups

### Intervention adherence

The attendance rate was 393/432 (91%) and 441/480 (92%) for AE and RE respectively. The most common reason given for missing a session was family or social commitments (28%). The completion rate for the additional home-based session during the last 6 weeks of the intervention was lower at 69% and 60% for AE and RE respectively (64% for the combined group). Adherence to the exercise prescription was 80% and 76% for AE and RE respectively. During RT (n = 16 only), attendance was 96% in AE and 95% in RE, adherence to the exercise prescription was 83% in AE and 75% in RE. Where an adjustment to the planned session prescription was required that results in a decrease in volume or intensity, 59% had the resistance element of the session altered, 26% had the aerobic element altered and 15% had both the aerobic and resistance element of the session altered. The most frequently needed modification involved a decrease in prescribed intensity, accounting for 75% of alterations. Participants undertook one low intensity/high volume and one high intensity/low volume session weekly. Of the sessions modified, 46% were low intensity/high volume sessions and 54% were high intensity/low volume sessions. Detailed information on adherence can be found in Table 2. Exercise-relative dose intensity (ExRDI) was also calculated for the resistance element of the programme [45]. The prescribed minimal total cumulative dose across the intervention was 203,412 ± 32,512 kg for RE and 61,162 ± 8,997 kg for AE. The completed total cumulative dose was 188,892 ± 39,152 kg for RE and 56,245 ± 10,202 kg for AE. This yielded an ExRDI of 93% ± 6% for RE and 92% ± 9% for AE.

**Table 2:** Reasons for modifications from the planned session prescription broken down by number of participants affected and number of sessions altered

Variable/Reasons	Participants *n* (%^b^)^a^			Sessions *n* (%^b^)		
	AE (*n* = 9)	RE (*n* = 10)	Total (*n* = 19)	AE	RE	Total
Non-attendance/Missed sessions						
**Health related**						
* Treatment related side effects*	0 (0%)	0 (0%)	0 (0%)	0 (0%)	0 (0%)	0 (0%)
* Feeling unwell*	4 (44%)	3 (30%)	7 (37%)	12 (30%)	4 (10%)	16 (20%)
* Hospital appointments*	4 (44%)	4 (40%)	8 (42%)	5 (13%)	9 (23%)	14 (18%)
* Covid*	1 (11%)	1 (10%)	2 (11%)	3 (8%)	3 (8%)	6 (8%)
**Non-health related**						
* Work commitments*	0 (0%)	2 (20%)	2 (11%)	0 (0%)	4 (10%)	4 (5%)
* Family/social commitments*	5 (56%)	5 (50%)	10 (53%)	12 (30%)	10 (26%)	22 (28%)
* Weather*	1 (11%)	3 (30%)	4 (21%)	1 (3%)	3 (8%)	4 (5%)
* Transport issues*	1 (11%)	3 (30%)	4 (21%)	2 (5%)	5 (13%)	7 (9%)
* No reason given*	1 (11%)	0 (0%)	1 (5%)	4 (10%)	0 (0%)	4 (5%)
* Other*	1 (11%)	1(10%)	2 (11%)	1 (3%)	1 (3%)	2 (3%)
**Total number of participants or sessions affected**	**8 (89%)**	**9 (90%)**	**17 (89%)**	**40**	**39**	**79**
**Exercise interruption**^**1**^						
**Health related**						
* Treatment related side effects*	0 (%)	0 (0%)	0 (0%)	0 (0%)	0 (0%)	0 (0%)
* Covid*	1(11%)	1 (10%)	2 (11%)	4 (19%)	3 (43%)	7 (33%)
* Medical procedure*	0 (0%)	1 (10%)	1 (5%)	0 (0%)	4 (57%)	4 (19%)
* Other*	1(11%)	0 (0%)	1 (5%)	7 (50%)	0 (0%)	7 (33%)
**Non-health related**						
* Holidays*	1 (11%)	0 (0%)	1 (5%)	3 (21%)	0 (0%)	3 (14%)
**Total number of participants or sessions affected**	**2 (22%)**	**2 (20%)**	**4 (21%)**	**14**	**7**	**21**
**Programme paused **^**2**^						
**Health related**						
* Blood pressure issues*	0 (0%)	1 (10%)	1 (5%)	0 (0%)	5 (100%)	5 (31%)
**Non-health related**						
* Holidays*	2 (22%)	0 (0%)	2 (11%)	9 (82%)	0 (0%)	9 (56%)
* Family commitments*	1 (11%)	0 (0%)	1 (5%)	2 (18%)	0 (0%)	2 (13%)
**Total number of participants or sessions affected**	**2 (22%)**	**1 (10%)**	**3 (16%)**	**11**	**5**	**16**
**Session modification**^3^						
**Health related**						
* Unwell (generalised)*	6 (67%)	4 (40%)	10 (53%)	12 (16%)	7 (7%)	19 (10%)
* Treatment related side effects*	6 (67%)	5 (50%)	11 (58%)	9 (12%)	8 (8%)	17 (9%)
* Delayed onset muscle soreness*	1 (11%)	2 (20%)	3 (16%)	1 (1%)	4 (4%)	5 (3%)
* Arthritis*	1 (11%)	3 (30%)	4 (21%)	15 (19%)	33 (31%)	48 (26%)
* Fear/Anxiety*	3 (33%)	2 (20%)	5 (26%)	4 (5%)	4 (4%)	8 (4%)
* Preexisting comorbidities*	2 (22%)	1(10%)	3 (16%)	4 (5%)	1 (1%)	5 (3%)
**Non-health related**						
* Returning to work/manual labour*	2 (22%)	3 (30%)	5 (26%)	2 (3%)	6 (6%)	8 (4%)
* Time constraints*	0 (0%)	4 (40%)	4 (21%)	0 (0%)	14 (13%)	14 (8%)
* Unable to maintain intensity*	8 (89%)	7 (70%)	15 (79%)	26 (34%)	23 (22%)	49 (27%)
* Other*	2 (22%)	4 (40%)	6 (32%)	2 (3%)	4 (4%)	6 (3%)
**Early session termination**						
* Dizziness*	1 (11%)	0 (0%)	1 (5%)	1 (1%)	0 (0%)	1 (0.5%)
* Nausea*	1 (11%)	0 (0%)	1 (5%)	1 (1%)	0 (0%)	1 (0.5%)
* Time constraints*	0(0%)	1(10%)	1 (5%)	0(0%)	2(2%)	2 (1%)
**Total number of participants or sessions affected**	**9 (100%)**	**10 (100%)**	**19 (100%)**	**77**	**106**	**183**

^1^Exercise interruption, missing at least three consecutive supervised treatments

^2^Programme paused, programme electively paused but missed sessions added to end of the programme

^3^Session modification, at least one element of a session required a reduction in exercise dose

^a^Number of reasons/variables sums to greater than the total number of participants listed because several participants required modifications for different reasons on different session days

^b^Numbers may not add up to 100% in each section because of rounding

### Suitability of the testing protocols

Six participants do not have pre- and post-intervention results for the modified Bruce treadmill test, four were unable to undertake treadmill testing due to balance, co-ordination and treadmill anxiety issues, technical issues were experienced in two tests. Three participants were unable to perform the sit and reach test due to orthopaedic issues. Five CRP measures were removed from that dataset due to very high values at the post-intervention timepoint indicative of acute inflammation post-radiation therapy. Four participants did not complete the EORTC QLQ-PR25 questions on sexual activity.

### Adverse events

No serious adverse events (grade 2 or higher) attributable to the exercise intervention were reported. Three grade 1 events were reported in the aerobic group. One participant became dizzy during an exercise session. Subsequent investigation uncovered that the participant attended the session fasted and dehydrated. Two participants had minor falls in the gym with no resultant injuries and no intervention required. All three participants continued with and completed the intervention without incident.

### Outcome measures

#### AE vs RE group changes

The response to the AE and RE intervention differed for some outcome measures. There were changes favouring AE in SF-36 physical function (*p* = 0.02) and role limitation (*p* = 0.01) values and also in haemoglobin (*p* < 0.05) (Table 3). There was a difference approaching significance (*p* = 0.06) in favour of AE for lower extremity lean soft tissue mass (Table 3). The BESS balance change scores were in favour (*p* = 0.05) of the RE group (Table 3). There were no other between group differences in any physical function, physical activity, vascular health, haematological or quality of life measure (Table 3).

**Table 3:** Change in the aerobic emphasised group (*n* = 9) relative to the resistance emphasised group (*n* = 10). Intervention effects on fitness, body composition, vascular, haematological, metabolic and quality of life outcomes, in the combined group (*n* = 19) at the pre-intervention, interim and post-intervention timepoints

Variable	Pre AE (*n* = 9) and RE (*n* = 10)	Δ AE (*n* = 9) and RE (*n* = 10)	*P* value Δ AE vs RE	Pre (*n* = 19)	Interim (*n* = 19)		Post (*n* = 19)	*P* value pre vs post
Physical Fitness								
Aerobic Fitness								
Six-minute walk test (m)	AE: 573 (77) RE: 514 (99)	AE: 38.5 (39.2) RE: 64.1 (34.0)	0.16	540.6 (92.7)			593.4 (97.5)	** < 0.01**
Modified Bruce submaximal treadmill test (secs)	AE: 619 (66) RE: 608 (180)	AE: 31 (69) RE: 30 (159)	0.98	613.6 (129.5)	618.5 (109.5)		643.9 (88.5)	0.39
Strength and Muscular Endurance								
Grip (right-left average, kg)	AE: 37.2 (9.3) RE: 37.7 (7.5)	AE: −0.4 (3.8) RE: −0.9 (2.9)	0.74	37.5 (8.2)	37.6 (8.1)		36.8 (6.8)	0.40
Leg press (kg)	AE: 80.3 (16.1) RE: 85.8 (15.6)	AE: 15.9 (10.3) RE: 39.5 (45.1)	0.14	83.2 (15.6)			110.9 (43.9)	** < 0.01**
Chest press (kg)	AE: 51.1 (16.6) RE: 60.5 (15.4)	AE: 1.4 (9.1) RE: 4.6 (15.0)	0.59	56.1 (16.3)			59.2 (15.8)	0.29
Isokinetic extension strength (right-left average, Nm)	AE: 144.9 (37.6) RE: 135.5 (42.8)	AE: 4.7 (15.8) RE: 13.3 (11.6)	0.19	139.9 (39.6)			149.2 (41.1)	**0.02**
Isokinetic flexion strength: (right-left average, Nm)	AE: 66.5 (23.9) RE: 68.1 (16.1)	AE: 5.6 (15.3) RE: 8.5 (7.9)	0.62	67.3 (19.6)			74.4 (14.8)	**0.02**
30 s sit to stand (reps)	AE: 13.0 (2.6) RE: 12.8 (3.2)	AE: 4.0 (1.7) RE: 5.7 (4.6)	0.31	12.9 (2.8)	16.0 (3.9)*		17.8 (5.6)	** < 0.01**
Mobility, Balance and Flexibility								
Timed up and go (secs)	AE: 8.0 (2.0) RE: 7.7 (1.3)	AE: −1.8 (1.0) RE: −1.2 (1.1)	0.27	7.8 (1.6)	6.7 (1.5)*		6.3 (1.6)	** < 0.01**
BESS balance test (errors)	AE: 23.6 (7.6) RE: 28.5 (10.2)	AE: 2.4 (6.7) RE: −6.0 (10.7)	**0.05**	26.2 (9.2)	23.8 (7.7)		24.1 (8.8)	0.37
Sit and reach (cm)	AE: 5.6 (4.5) RE: 9.5 (9.8)	AE: 0.7 (2.2) RE: 1.5 (2.7)	0.35	7.7 (7.8)	8.1 (7.5)		8.8 (7.6)	0.10
Physical Activity								
Godin Leisure score	AE: 7.5 (8.1) RE: 6.8 (7.6)	AE: 17.1 (3.9) RE: 15.9 (6.0)	0.63	7.1 (7.6)			23.6 (9.1)	** < 0.01**
Body Composition								
Lean soft tissue mass (g)	AE: 53,125 (8495) RE: 55,753 (6663)	AE: 611 (2035) RE: −1052 (2794)	0.16	54,508 (7489)			54,242 (7270)	0.66
Upper extremity lean soft tissue mass (g)	AE: 6059 (1224) RE: 6859 (1350)	AE: 15.7 (354.1) RE: −247 (406.7)	0.15	6480 (1321)			6357 (1252)	0.19
Lower extremity lean soft tissue mass (g)	AE: 16,492 (3155) RE: 17,247 (1908)	AE: 205.7 (715.5) RE: −523.2 (841.8)	0.06	16,889 (2529)			16,712 (2482)	0.37
Lean soft tissue index (kg/m^2^)	AE: 7.5 (1.0) RE: 8.3 (0.7)	AE: 0.06 (0.4) RE: −1.1 (2.8)	0.26	7.9 (0.9)			7.8 (0.9)	0.31
Total fat mass (g)	AE: 25,814 (8727) RE: 27,871 (6434)	AE: 1839 (1870) RE: 807 (2063)	0.27	26,897 (7461)			28,193 (6710)	**0.01**
VAT area (cm^2^)	AE: 216 (97) RE: 239 (47)	AE: 3.2 (32.1) RE: −8.9 (17.3)	0.31	228 (73.4)			225 (68.4)	0.59
Weight (kg)	AE: 82.4 (16.0) RE: 87.2 (12.2)	AE: 2.3 (2.8) RE: −0.4 (3.7)	0.09	84.9 (13.9)			85.8 (13.5)	0.29
Vascular Markers								
Systolic blood pressure (mmHg)	AE: 137.6 (12.9) RE: 137.1 (12.9)	AE: −7.6 (11) RE: −3.8 (7.6)	0.41	137.3 (12.6)			131.7 (12.0)	**0.02**
Diastolic blood pressure (mmHg)	AE: 80.0 (4.2) RE: 80.7 (9.0)	AE: −4.3 (5.9) RE: −2.4 (4.4)	0.43	80.4 (7.0)			77 (6.2)	**0.01**
Pulse-wave velocity (m/s)	AE: 12.6 (2.3) RE: 10.6 (1.2)	AE: 0.29 (0.99) RE: 0.26 (1.2)	0.99	11.9 (2.2)			12.1 (2.1)	0.35
Haematological Markers								
White blood cell count (10^3^/µL)	AE: 6.4 (1.7) RE: 5.7 (2.0)	AE: −0.06 (1.7) RE: −1.4 (1.6)	0.09	6.0 (1.8)			5.3 (1.7)	0.07
Red blood cell count (10⁶/µL)	AE: 4.5 (0.2) RE: 4.6 (0.4)	AE: −0.2 (0.3) RE: −0.4 (0.3)	0.13	4.6 (0.3)			4.2 (0.3)	** < 0.01**
Haemoglobin (g/dL)	AE: 13.8 (0.6) RE: 14.3 (1.1)	AE: −0.5 (0.6) RE: −1.3 (0.8)	**0.04**	14.1 (0.9)		13.1 (0.9)		** < 0.01**
Hematocrit (%)	AE: 41.4 (1.5) RE: 42.0 (3.2)	AE: −1.6 (2.4) RE: −3.5 (2.5)	0.11	41.7 (2.5)			39.1 (2.5)	** < 0.01**
Metabolic Markers								
Cholesterol (mmol/L)	AE: 4.7 (0.3) RE: 5.3 (1.3)	AE: −0.2 (0.9) RE: −0.1 (0.7)	0.79	5.0 (1.0)			4.8 (1.1)	0.29
HDL cholesterol (mmol/L)	AE: 1.41 (0.56) RE: 1.25 (0.29)	AE: 0.0 (0.3) RE: 0.0 (0.2)	0.79	1.32 (0.4)			1.35 (0.5)	0.67
LDL cholesterol (mmol/L)	AE: 2.8 (0.7) RE: 3.3 (1.0)	AE: −0.3 (0.6) RE: −0.2 (0.5)	0.94	3.0 (0.9)			2.8 (0.9)	0.08
Triglycerides (mmol/L)	AE: 1.08 (0.30) RE: 1.56 (0.71)	AE: −0.0 (0.4) RE: −0.0 (0.4)	0.92	1.33 (0.6)			1.31 (0.6)	0.86
Glucose (mmol/L)	AE: 5.0 (0.3) RE: 5.1 (0.5)	AE: 0.2 (0.7) RE: 0.1 (0.6)	0.82	5.1 (0.5)			5.2 (0.5)	0.31
Insulin (mIU/L)	AE: 8.3 (4.4) RE: 9.7 (5.5)	AE: 0.28 (1.7) RE: 0.31 (3.7)	0.98	9.1 (5.0)			9.4 (3.5)	0.67
HOMA-IR	AE: 1.87 (1.07) RE: 2.27 (1.49)	AE: 0.14 (0.38) RE: 0.03 (1.12)	0.79	2.10 (1.3)			2.18 (0.8)	0.69
C-reactive protein (mg/L)	AE: 1.8 (1.2) RE: 2.8 (2.1)	AE: 0.5 (0.7) RE: −0.8 (2.1)	0.15	2.3 (1.7)			2.2 (1.5)	0.86
Quality of Life and Fatigue								
FACT-F	AE: 40.9 (8.6) RE: 47.6 (3.3)^a^	AE: 0.3 (7.1) RE: −2.9 (7.0)	0.33	44.4 (7.1)	44.6 (5.9)		43.1 (7.3)	0.41
EORTC QLQ-PR25								
Urinary symptoms	AE: 21.0 (16.4) RE: 14.2 (9.2)	AE: 1.2 (8.8) RE: 0.9 (9.3)	0.92	17.4 (13.2)	23.5 (13.7)*		18.4 (13.5)	0.61
Bowel symptoms	AE: 6.5 (7.0) RE: 2.5 (4.0)	AE: −1.9 (9.1) RE: 6.7 (11.6)	0.09	4.4 (5.8)	4.4 (7.0)		7.0 (9.7)	0.32
Hormonal related symptoms	AE: 16.7 (12.7) RE: 4.4 (5.1)	AE: 4.8 (14.8) RE: 7.2 (13.3)	0.71	10.2 (11.1)	13.7 (10.0)		16.3 (11.6)	0.07
Sexual activity	AE: 19.0 (26.2) RE: 14.6 (13.9)	AE: −9.6 (41.5) RE: −12.5 (14.8)	0.85	16.6 (19.9)	7.7 (22.5)		5.5 (17.3)	0.16
EORTC QLQ-C30								
Global health status	AE: 65.6 (17.8) RE: 87.4(14.3)^a^	AE: 5.4 (20.3) RE: −1.9 (10.8)	0.33	77.7 (19.6)	78.9 (17.5)		79.0 (20.2)	0.74
Physical function	AE: 81.9 (20.2) RE: 96.0 (7.1)^a^	AE: 2.6 (19.7) RE: −1.0 (3.2)	0.58	89.3 (16.1)	93.3 (10.6)		90.0 (12.7)	0.82
Role function	AE: 79.6 (28.5) RE: 98.3 (5.4)^a^	AE: 9.2 (23.9) RE: −4.9 (11.1)	0.11	89.5 (21,6)	92.2 (17.7)		91.3 (16.0)	0.69
Emotional function	AE: 80.6 (12.6) RE: 93.3 (9.5)^a^	AE: 4.9 (7.0) RE: 0.9 (10.5)	0.35	86.5 (12.5)	86.0 (14.5)		89.6 (13.1)	0.19
Cognitive function	AE: 81.4 (22.8) RE: 84.8 (14.6)	AE: 1.9 (27) RE: 1.8 (12.4)	0.99	83.2 (19.0)	82.4 (15.5)		85.2 (16.0)	0.69
Social function	AE: 88.9 (18.6) RE: 98.3 (5.4)	AE: −1.8 (22.7) RE:0	0.81	93.5 (14.2)	89.8 (19.8)		92.6 (15.3)	0.82
Fatigue	AE: 23.3 (19.6) RE: 12.1 (11.0)	AE: 0.6 (14.9) RE: 2.1 (15.4)	0.83	17.4 (16.2)	18.0 (12.3)		18.8 (15.5)	0.69
Nausea/vomit	AE: 1.9 (5.7) RE: 0	AE: 0 (8.5) RE: 0	1	0.9 (3.9)	0 (0)		0.9 (3.9)	1.0
Pain	AE: 27.8 (35.3) RE: 6.8 (8.7)	AE: −5.4 (37.2) RE: −1.8 (16.7)	0.78	16.7 (26.6)	13.2 (21.2)		13.2 (21.3)	0.59
Dyspnea	AE: 29.2 (37.6) RE: 6.6 (13.9)	AE: −12.4 (17.1) RE: 0 (22)	0.21	16.6 (28.6)	5.6 (12.6)		11.1 (22.9)	0.27
Insomnia	AE: 33.3 (37.3) RE: 9.9 (15.9)	AE: 0 (29) RE: 0 (22)	1	21.0 (29.9)	29.7 (24.6)		21.0 (29.9)	1.0
Appetite loss	AE: 11.1 (23.6) RE: 6.6 (14)	AE: 0 (16.5) RE: −6.6 (14)	0.36	8.7 (18.7)	3.5 (15.3)		5.3 (22.9)	0.33
Constipation	AE: 0 (0) RE: 13.3 (23.3)	AE: 3.7(11) RE: −6.7 (21)	0.20	7.0 (17.9)	3.5 (10.4)		5.2 (12.4)	0.66
Diarrhoea	AE: 7.4 (22.3) RE: 0	AE: −0.1 (16.8) RE: 3.3 (10.4)	0.60	3.7 (15.8)	9.3 (27.6)		5.5 (12.7)	0.59
Financial difficulties	AE: 3.7 (11.0) RE: 3.3 (10.5)	AE: 0 (16.5) RE: 3.3 (18.7)	0.69	3.5 (10.5)	7.4 (14.2)		5.5 (12.7)	0.67
SF-36								
Physical function	AE: 66.1 (23.8) RE: 82.4 (17.3)	AE: 12.2 (15.4) RE: −2.1 (7.6)	**0.02**	75.5 (22.1)	83.3 (21.2)		81.0 (18.9)	0.11
Role limitation (physical health)	AE: 60.2 (39.5) RE: 90.0 (21.1)^a^	AE: 12.0 (28.9) RE: −25.0 (26.4)	**0.01**	75.8 (33.9)	71.1 (31.5)		68.4 (37.1)	0.34
Role limitation (emotional Problems)	AE: 70.4 (42.3) RE: 96.7 (10.5)	AE: 22.2 (44.1) RE: −3.3(18.9)	0.11	84.2 (32.1)	86.0 (27.9)		93.0 (13.9)	0.29
Energy/fatigue	AE: 54.4 (13.3) RE: 76.4 (10.5)^a^	AE: 8.9 (15.2) RE: −2.9 (22.3)	0.20	66.0 (16.2)	68.4 (16.0)		68.7 (19.7)	0.56
Emotional well-being	AE: 76.0 (13.3) RE: 88.4 (7.2)^a^	AE: 2.2 (15.9) RE: 3.2 (11.9)	0.88	82.5 (12.0)	86.3 (12.2)		85.3 (16.1)	0.39
Social function	AE: 79.2 (22.5) RE: 95.0 (8.7)^a^	AE: 8.3 (17.7) RE: −2.5 (12.9)	0.14	87.5 (18.2)	86.8 (18.9)		90.1 (14.8)	0.48
Pain	AE: 66.4 (27.4) RE: 83.8 (18.6)	AE: 10.8 (20.1) RE: −1.7 (21.9)	0.21	76.1 (24.8)	82.1 (21.7)		79.2 (27.1)	0.55
General health	AE: 61.8 (19.4) RE: 82.1 (19.8)^a^	AE: 11.4 (17.4) RE: −1.5 (15.8)	0.11	72.4 (22.3)	72.6 (23.8)		78.6 (21.0)	0.12

Data expressed as mean (SD)

*p < 0.05 at the interim timepoint compared to pre-intervention

^a^*p* < 0.05 compared to AE at pre-intervention

***Δ*** Delta (change), *BESS* Balance error scoring system, *FACT-F* The Functional Assessment of Cancer Therapy-Fatigue, *EORTC QLQ* European Organisation for the Research and Treatment of Cancer Quality of Life Questionnaire, *SF-36* Medical Outcomes Short Form 36, *VAT* Visceral adipose tissue, *HDL* High density lipoprotein, *LDL* Low density lipoprotein, *HOMA-IR* Homeostatic Model Assessment of Insulin Resistance. *Lean soft tissue index* Lean soft tissue (kg)/height^2^ (m)

All *n* = 19 except; Six-minute walk test *n* = 18, Modified Bruce treadmill test *n* = 12, Sit and reach *n* = 15, EORTC QLQ-PR25 (sexual activity) *n* = 15, EORTC QLQ-C30 (Global health status, emotional function, cognitive function, dyspnea, diarrhoea, financial difficulties) *n* = 18, SF-36 (Physical function, pain, general health) *n* = 18, Pulse-wave velocity *n* = 12, Glucose *n* = 18, Insulin *n* = 18, C-reaction protein, *n* = 13, HOMA-IR *n* = 18. Interim data collection included a select number of outcome variables. There were insufficient responses in domains relating to incontinence aids and sexual function in EORTC QLQ-PR25 so they have been excluded from the analysis

#### Combined group pre- to post-intervention analysis

Improvements were observed in physical fitness between pre- and post-intervention for the combined AE and RE groups (*n* = 19). There were improvements in the six-minute walk test (Cohen’s d = 1.4), STS (Cohen’s d = 1.4), TUG (Cohen’s d = −1.4), sit and reach test (Cohen’s d = 0.5), leg press 1RM (Cohen’s d = 0.8), isokinetic peak torque assessments (Cohen’s d ~ 0.6) and physical activity levels via the Godin Leisure Time Exercise Questionnaire (Cohen’s d = 3.3). Pre- to post-intervention reductions (*p* < 0.05) in systolic (Cohen’s d = −0.6) and diastolic (Cohen’s d = −0.65) blood pressure were observed, but no change in pulse wave velocity. Despite the exercise intervention, pre- to post-intervention decreases (*p* < 0.05) in red blood cell count, haemoglobin and haematocrit were observed. Despite the exercise intervention, there was an increase in total fat mass, with no change in any index of lean soft tissue mass (Table 3) There were no pre- to post-intervention differences for any quality of life domains or fatigue (based on SF-36, EORTC QLQ C30, EORTC QLQ PR-25 or FACT-F) or any metabolic health markers (Table 3).

#### Interim assessments

Interim assessments were undertaken for selected outcomes. In the case of outcomes for which there was no pre- to post-intervention difference, the interim value was not different to either the pre- or post-intervention value. In the case of the TUG and STS functional fitness tests there was a significant improvement at the interim timepoint from pre-intervention that was maintained at post-intervention (Table 3).

### Test- retest correlation coefficients and sample size estimates

Combined group test – retest correlation coefficients for key outcome variables were as follows; lean body mass, r = 0.94; fat mass, r = 0.97; appendicular skeletal muscle index, r = 0.88; systolic blood pressure, r = 0.71; pulse wave velocity, r = 0.89; 6-min-walk, r = 0.92; right hand grip strength, r = 0.90; timed-up-and-go test, r = 0.77; sit-to-stand test, r = 0.80; FACT-F, r = 0.52. We estimate that the number of participants required for each arm of a future larger trial would be as follows (outcome measure, change required, participants per arm): systolic blood pressure, 4 mmHg, *n* = 71; lean body mass, 1 kg, *n* = 95; fat mass, 1 kg, *n* = 47; FACT-F, 4 units, *n* = 45.

## Discussion

This is the first study to compare an aerobic-emphasised to a resistance-emphasised exercise intervention in men with prostate cancer. The primary focus of the study was feasibility, the feasibility of comparing two complex mixed mode and mixed intensity prescriptions that were being progressed over a 24-week intervention period in conjunction with both ADT and RT treatments. The results demonstrate good retention, adherence and safety in both trial arms. However, recruitment was challenging with a rate lower than anticipated.

Recruitment has been a long-cited issue in clinical trials [63]. Despite embedding a direct referral pathway from the radiation oncologist to the trial co-ordinator, the recruitment rate remained low at 19%, primarily due to the travel requirements to the exercise centre. Though challenging, this rate is not dissimilar to other exercise oncology trials [64–66], where travel has also been cited as a barrier to participation [64, 66]. Particularly pertinent for this trial perhaps was the relatively small catchment population (*circa* 80,000) within a 30 min drive from the exercise centre. Additional exercise centres, possibly satellite centres and recruitment hospitals would be needed before progressing to a definitive trial. Based on our sample sizes estimates, 150–300 participants may be needed for a future three arm definitive trial, depending on the primary outcome variable of interest.

Participant characteristics were as expected, with the majority having additional comorbidities [67, 68]. The prevalence of hypertension, other cardiovascular issues and arthritis have implications for safety. Future trials involving high intensity exercise in this cohort group would therefore be advised to plan for these cardiovascular co-morbidities in terms of instructor expertise and ongoing medical oversight. Despite these challenges, 75% of those assessed initially were eligible for randomisation which is a positive feasibility finding.

Retention, attendance and prescription adherence was excellent in both trial arms, at least on a par with that reported in similar trials [66, 69, 70]. Attendance at exercise sessions was over 90%. Attendance during RT was similarly high in both the AE and RE groups. This is re-assuring for future trials but also understandable in part given that the primary reason cited for missed sessions was family or social commitments. No sessions were missed due to treatment side effects. Radiation therapy hospital treatment visits may in fact be a facilitator rather than a barrier to exercise participation. The intervention was also acceptable to participants, the results of which are reported elsewhere [47].

A broad range of outcome measures were successfully assessed with a few exceptions. With hindsight, an incremental cycle ergometer may have been more appropriate for this population given the orthopaedic limitations and treadmill co-ordination issues. A maximal test with appropriate supervision may also have been more sensitive to changes in aerobic fitness. In addition, it may not be appropriate to employ inflammatory markers as indicators of chronic low-grade inflammation in the weeks following completion of RT.

The key objective of this study was to determine the feasibility of undertaking this particular comparison of an aerobic- with a resistance-emphasised protocol which included high and moderate intensities of aerobic and resistance exercise within each arm. To this end, we closely monitored daily adherence to the exercise prescription and adverse events for all participants. Exercise prescription adherence was high overall with little difference between trial arms. Prescription adherence remained high during RT, slightly higher in the AE group. This is particularly impressive as high intensity aerobic exercise as well as high percentage hypertrophy resistance exercise were part of the prescription. Comparison of the interim and post-intervention assessments (interim assessments undertaken just before the start of RT) provide further evidence that physical function changes and do not regress during RT. The feasibility of these exercise protocols during RT is an important finding of this study. With the exception of three grade 1 events, there were no serious adverse events in either arm. Overall, the resistance element was more likely than the aerobic element to need modification, with arthritic joints identified as the primary factor contributing to these modifications. Comorbidities unrelated to prostate cancer or its treatment have been highlighted previously as the main reason for exercise alterations in prostate cancer cohorts [71]. Completion of the home sessions (i.e. attendance) was lower in both trial arms. This would suggest that greater monitoring and attention is needed on any home-based element when progressing to a definitive trial. Activity monitors may be warranted and could add additional insights into which exercise prescription leads to higher physical activity outside of the supervised sessions.

We are aware of three previous studies that have compared supervised exercise modalities in individuals with prostate cancer undergoing treatment [26–28]. Although this study was not adequately powered to test for differences between the two intervention arms, there are some preliminary differences that warrant comment. Favouring aerobic-emphasised training there were between group differences in the SF-36 scores for physical function and physical health role limitation. This contrasts with previous literature which reported additional benefits for quality of life from resistance exercise [26]. Two other studies have suggested no difference between aerobic exercise and resistance exercise for quality of life [27, 28]. The SF-36 results observed must also be considered in the context of unusually high pre-intervention values in the RE group but also some pre-intervention quality of life differences between AE and RE. It is not possible to stratify for all outcome measures, but stratification by quality of life may be necessary in future studies where it is the primary outcome measure.

In line with previous reports [72, 73], there was a treatment-related reduction in white blood cells, red blood cell count, haemoglobin and haematocrit. The results of this study suggest that aerobic-emphasised exercise may counteract against this reduction, at least for haemoglobin. To our knowledge this is the first report of the potential of any exercise intervention to impact on the reduction in haematological variables during ADT and RT treatment and further study is warranted. Previous studies [26, 74], that report on these variables did not show any mitigation from the more moderate intensity exercise training regimes described. The aerobic-emphasised exercise prescription employed in this study which combined high intensity interval exercise and higher volumes of moderate intensity continuous exercise may have provided a greater stimulus for erythropoiesis. Favouring resistance-emphasised exercise, there was a between group difference in BESS balance scores. As individuals treated with ADT have increased risk of fracture compared to those not treated with ADT [75], improvement in balance scores represent an important adaptation to training.

From this limited dataset, it appears that both exercise regimes were equally effective at increasing performance in the 6-min walk test and isokinetic strength assessments. The issues with the incremental treadmill test potentially limited a valid comparison of each regime on aerobic fitness. Should it be a primary outcome, future studies need to consider how best to assess aerobic fitness in this cohort, to include the possibility of personalised approaches and maximal tests. The body composition comparisons yielded unexpected results. Though no significant between group difference was observed, the change in lower extremity lean body mass, if anything, tended to favour aerobic-emphasised exercise, with no evidence that resistance-emphasised exercise was superior for increasing any index of lean body mass. There was a need to adjust the resistance element of session due to arthritis in some individuals which may have been a factor in this small study. The results however raise the possibility of benefits to lean tissue in those on ADT from even small volumes of resistance exercise combined with higher volumes of aerobic exercise, some at high intensity. The maintenance of lean tissue is particularly pertinent in a cohort initiating ADT treatment, due to the possible role of myokines in the suppression of prostate tumour growth and metastases [76].

Although the primary planned analysis involved a comparison of the effects of AE vs RE on multiple outcome measures, there were some interesting observations from the comparison of the pre- and post-intervention values in the combined AE and RE group. There were improvements in the 6-min walk test, STS, TUG, sit and reach test, leg press 1RM and isokinetic strength assessments at the post-intervention timepoint. Decreases in systolic and diastolic blood pressure were also observed but no change in any of the cardiometabolic blood markers. There was an increase in fat mass, consistent with previous reports of body fat changes on ADT [77, 78]. No changes were observed in the combined group fatigue or quality of life scores despite the multiple assessment tools employed. It should be noted however that the majority of participants recruited were at the initiation of their ADT treatment and before RT, with baseline scores not showing clinical levels of fatigue or suboptimal quality of life. The improvement in multiple outcome measures post-intervention, despite ongoing ADT and RT treatment, is evidence of exercise efficacy and in line with the existing evidence base [10–15].

### Limitations

The absence of a control group is the key limitation in this study. Even though many of the benefits of exercise for prostate cancer have been established, it is still desirable to employ a non-exercise control group in any exercise study that commences near the start of ADT when detrimental changes in body composition, fitness, haematological variables and quality of life might be expected. Trial commencement by some participants after RT is another limitation, though these represent only a small proportion of those who completed (3/19). The absence of objective monitoring of moderate and high intensity aerobic exercise is another limitation, future studies comparing exercise modalities should strive to record adherence to the aerobic element of the programme in addition to the resistance element which is reported using the exercise relative dose intensity approach. Issues with the incremental submaximal treadmill test in some participants is a further limitation that may have resulted in an underestimation of aerobic fitness gains in one or both arms.

## Conclusion

In conclusion, intervention trials that carefully structure aerobic and resistance-emphasised protocols and that progressively overload both components, are feasible for men with prostate cancer undergoing active treatment, with evidence from this feasibility trial of good retention, attendance, prescription adherence and safety and some evidence of efficacy. Continuation of such trials are also feasible during RT. There was some early evidence of differential effects from protocols that emphasise aerobic or resistance elements. Strategies would have to be implemented to increase recruitment however, including delivery at multiple sites in a definitive trial, with appropriate planning for the cardiovascular and orthopaedic co-morbidities likely to be observed. A definitive trial is justified.

## Data Availability

The datasets generated during and/or analysed during the current study are available from the corresponding author on reasonable request.
